# Associations between parental food choice motives, health-promoting feeding practices, and infants’ fruit and vegetable intakes: the Food4toddlers study

**DOI:** 10.29219/fnr.v64.3730

**Published:** 2020-10-12

**Authors:** Margrethe Røed, Frøydis Nordgård Vik, Elisabet Rudjord Hillesund, Wendy Van Lippevelde, Nina Cecilie Øverby

**Affiliations:** 1 Department of Nutrition and Public Health, Faculty of Health and Sports Sciences, University of Agder, PO box 422, 4604 Kristiansand, Norway; 2 Department of Marketing, Innovation and Organisation, Faculty of Economics & Business administration, Ghent University, Tweekerkenstraat 2, 9000 Ghent, Belgium

**Keywords:** infant, healthy food intake, mediation, diet

## Abstract

**Background:**

Parents influence their infants’ diets and are the providers of healthy foods such as fruit and vegetables. Parental motives can influence infant’s diets directly or through parental feeding practices.

**Objective:**

This study aimed to assess the associations between parental food choice motives and infants’ fruit and vegetable intakes and to examine whether parental feeding practices mediated these associations.

**Design:**

A total of 298 parents participated in the Norwegian Food4toddlers study. Before the child’s first birthday (mean age = 10.9 months), the parents completed an online baseline questionnaire. Five parental food choice motives were assessed: health, convenience, sensory appeal, price, and familiarity. Infants’ fruit and vegetable intakes and three health-promoting feeding practices were also assessed. For each food choice motive and its relation to fruit or vegetable intake, three single mediation models were conducted. Mediation effects were examined using MacKinnon’s product of coefficients procedure, and bootstrap confidence intervals (CIs) were used for inferential testing.

**Results:**

Higher scores on the motive of health were positively associated with infants’ vegetable intake (*τ* = 0.394, *P* < 0.001). No other significant associations were found between food choice motives and fruit or vegetable intake. The feeding practice of shaping a healthy environment mediated the relationships between health motive and both fruit (*αβ* = 0.067, CI: 0.001–0.146) and vegetable (*αβ* = 0.105, CI: 0.042–0.186) intakes. The feeding practice of encouraging balance and variety mediated the relationships between health motive and vegetable (*αβ* = 0.085, CI: 0.030–0.150) intake and between sensory appeal motive and vegetable intake (*αβ* = 0.047, CI: 0.005–0.103).

**Conclusion:**

High levels of parental health motive are associated with higher infant vegetable intake. Our study contributes to understand the structure of parental feeding behaviors that may have implication for nutrition interventions targeting parents.

## Popular scientific summary

- The parental food choice motive of health is associated with higher infant vegetable intake.
- Health-promoting feeding practices mediate the relationships between the parental food choice motives of health and sensory appeal and their infants’ fruit and vegetable intakes, and the feeding practices of shaping the environment and encouraging balance and variety are the strongest mediators on these associations.
- The findings contribute to the understanding of parental feeding behaviors.

## 

Food is fundamental for health, growth, and development and also plays a central part in the increasing childhood obesity rates (1). Eating behaviors early in life track into later childhood and adult life (2–5). Efforts to establish a healthy diet should therefore start early (5, 6).

### Importance of fruit and vegetables

Fruit and vegetables are valuable sources for a wide range of micronutrients, fiber, and antioxidants and important for growth and development (7, 8). Further, a healthy diet rich in fruits and vegetables is known to prevent certain cancers and to reduce the risk of cardiovascular diseases, diabetes, and mortality (9, 10), and is considered as an important part of infants’ healthy dietary patterns and diet quality (11, 12). The infant period between 6 and 12 months, when solid food is recommended to introduce, is important for the development of the child’s food and eating behavior. Offering a variety of fruit and vegetables in this period may be especially important for a higher consumption in childhood (2, 13). Still, the fruit and vegetable intakes among infants in Norway reported in national surveys are suboptimal (5, 14).

### Parental feeding practices

Parents of infants play a key role in what their children eat. They provide food and shape the food and eating environment for their children (15–17). The infants are totally dependent on the adult according to how nutritious food they are provided. Parental feeding practices have been shown to be central in the development of obesogenic eating behaviors and excessive weight gain in young children (18). Relevant parenting practices include both intentional and unintentional behaviors and actions parents perform that influence their children’s attitudes, behaviors, or beliefs (19).

Several studies have focused on *coercive control practices* (also called negative feeding practices) and how they affect children (20, 21). Other dimensions, *structure* and *autonomy support and promotion*, entail practices that are positive and promote healthy eating among children, such as providing a healthy food and eating environment, encouraging balance and variety, and healthy modeling (19). These positive practices are of interest in this article.

Vaughn et al. (19) place food parenting practices in a large conceptual model, including how parents’ motives influence their food parenting practices and their children’s dietary intakes. Parental motives can influence a child’s dietary intake directly or indirectly through food parenting practices (19). When it comes to what kind of foods parents buy and serve their children, parents may be driven by different motives (e.g. purchasing inexpensive foods or pleasure). Most parents have a strong intention to both promote healthy eating and create a healthy food environment for their children, but there is a tendency for these good intentions to not necessarily translate into actual behavior (22, 23).

To our knowledge, few studies have explored how food choice motives act as precursors for parental feeding practices. Two studies conducted by Kiefner-Burmeister et al. (21) and Hoffmann et al. (24) investigated the effect of negative feeding practices on the association between maternal feeding motives and children’s diets, but health-promoting feeding practices have not yet been examined in relation to this association.

The aim of the present study was to examine the potential associations between parental food choice motives (health, convenience, sensory appeal, price, and familiarity) and infants’ fruit and vegetable intakes. Further, we aimed to examine the potential mediating effects of three health-promoting feeding practices (encouraging balance and variety, shaping a healthy environment, and healthy modeling) on these associations.

## Methods

### Procedure and participants

This study used baseline data from the Food4toddlers randomized controlled intervention study. Food4toddlers is a digital intervention aiming to promote healthy dietary habits among toddlers (12–18 months) (25). The recruitment period for this study was from August 2017 to January 2018, and our aim was to recruit 474 parent/infant dyads (25).

Parents of infants in Norway were recruited through tailored advertisement (i.e. targeting potential parental age and interest groups) on social media (Facebook). In Norway, 67% of the population uses Facebook daily (26). The Facebook advertisement included a relevant video or a picture and a link to the project website, where the parents received extended information about the intervention and had the opportunity to sign up. Consent for participation was obtained as part of the sign-up process. Participants had to be literate in Norwegian and have a child who was born from June 2016 to May 2017.

Approximately 1–2 months before the infant’s first birthday, those who signed up for the study received an email with a link to the baseline questionnaire. Data were collected using SurveyXact, an online survey software tool. The protocol for the present study was approved by the Norwegian Centre for Research Data (08/06/2016, reference 48,643) and is in accordance with the Helsinki Declaration of 1975, as revised in 2008.

We recruited 404 parents of infants through Facebook. One to two months before each infant turned 1 year old, a baseline questionnaire was sent to the parents. A total of 298 (response rate 73.8%) parents who originally signed up for the study answered more than half of the questions in the baseline questionnaire and were included in the present analyses.

Most participants were mothers (98.0%), and the mean age was 31.7 years (standard deviation [SD] = 4.2). Most parents lived in two-adult households (99.0%), 86.7% of the parents were born in Norway and the majority of participants were from Eastern Norway (43.3%), which has the densest population. See Table 1 for more details.

**Table 1:** Characteristics of participating parents and infants at baseline

Characteristics	Total
**Parents (N = 298)**	
Parent filling out the form: mother (%)	98.0
Age in years, mean (standard deviation [SD])^a^	31.7 (4.2)
Body mass index (BMI), mean (SD)^b^	25.0 (4.7)
Two-adult household (%)	99.0
Total number of household members, mean (SD)	3.6 (0.92)
Born in Norway (%)	86.7
**Education (%)^b^**	
Upper-level secondary school or less	11.7
College/university (≤4 years)	33.9
College/university (>4 years)	53.7
Other	0.7
**Geographic residence (%)**	
Northern Norway	6.0
Central Norway	10.7
Western Norway	21.8
Southern Norway	18.1
Eastern Norway (including Oslo)	43.3
**Children**	
Age in months, mean (SD)	10.9 (1.25)
Child’s sex: male (%)	55

a There was one missing case on this variable.

b There were two missing cases on this variable.

### Measures

Each participant reported their age, the age of the child, the number of persons in the household, the county of residence, and their own level of education. These items have previously been used and tested in Norway (14). The participants also reported whether Norway was the country of birth and their own body mass index (BMI) (self-reported).

### Independent variables: food choice motives

The Food Choice Questionnaire (FCQ) was used to assess parents’ motives underlying their selection of food. Developed by Steptoe et al. (27), the FCQ is widely used and has been tested in other context at country and cross-national levels (28, 29). For the present study, the questions were translated into Norwegian, back-translated into English, and adjusted as needed.

The FCQ comprises 36 items grouped into nine factors (health, mood, convenience, sensory appeal, natural content, price, weight control, familiarity, and ethical concerns), and responses to the original FCQ were on a four-point scale (27). Fotopoulos et al. (29) suggested using a seven-point scale to elicit a wider range of answers; this approach was used in the present study.

In the questionnaire, participants were asked to rate their level of endorsement of statements such as ‘It’s important to me that the food I eat on a typical day […]’, rating each statement from 1 (extremely unimportant) to 7 (extremely important) (29). The reliability of the factors used was tested using Cronbach’s alpha (*α*).

Five factors were used in this present study (Cronbach’s *α* values presented are for our sample): *health* (e.g. ‘It’s important to me that the food I eat on a typical day is high in protein’, six items, *α* = 0.81), *convenience* (e.g. ‘It’s important to me that the food I eat on a typical day is easy to prepare’, five items, *α* = 0.79), *sensory appeal* (e.g. ‘It’s important to me that the food I eat on a typical day looks nice’, four items, *α* = 0.64), *price* (e.g. ‘It’s important to me that the food I eat on a typical day is cheap’, three items, *α* = 0.73), and *familiarity* (e.g. ‘It’s important to me that the food I eat on a typical day is familiar’, three items, *α* = 0.73). These five factors were included in the baseline questionnaire for the Food4toddlers intervention because they were regarded as important precursors for the development of a healthy food and eating environment for toddlers. The Cronbach’s *α* values for this study were slightly lower than those reported by Pollard et al. (30) (except for *familiarity*) and higher for three out of five items (all items except *sensory appeal* and *price*) compared with the study of Fotopoulos et al. (29).

We did not perform a full-scale reproducibility study; however, in October 2018, the items were tested for reproducibility through a test–retest study at two time points (2 weeks apart) with 29 participating parents who did not participate in the intervention recruited from several local kindergartens. The standardized measure, Pearson’s correlation coefficient (*r*), showed acceptable-to-excellent correlations for the factors used (health: *r* = 0.910; convenience: *r* = 0.933; sensory appeal: *r* = 0.777; price: *r* = 0.846; familiarity: *r* = 0.726).

### Outcome variables: infants’ fruit and vegetable intakes

Infants’ fruit and vegetable intakes were assessed using the Food Frequency Questionnaire, which was previously used in a nationwide Norwegian diet survey among 12-month-old children (14). A validation study of the Food Frequency Questionnaire has been conducted for 1-year-old Norwegian children (31). In the questionnaire, parents report their infant’s frequency of consumption of fruits and vegetables. The questionnaire items include fresh, cooked, or squeezed fruits and vegetables, as well as both homemade and commercially produced variants.

These items are answered on a six-point scale ranging from *never* to *several times a day*. In the present study, the response options were recoded to reflect times per week: *never* or *less than once a week* = 0, *one to three times a week* = 2, *four to six times a week* = 5, *once a day* = 7, *twice a day* = 14, and *three times or more per day* = 24.5 (3.5 times/day was used in the calculation of this value). Similar recoding has previously been used by others (21, 32–34).

The items included fruits and vegetables normally consumed in Norway (e.g. apples, melons, carrots, and tomatoes), and there was also the additional item of ‘other fruits/vegetables’. The reported weekly consumption scores for these items were aggregated into sum scores and divided by seven. Results for fruits (11 items) and vegetables (13 items) showed the daily frequency of fruit and vegetable intakes.

### Potential mediating factors: parental health-promoting feeding practices

Parental feeding practices were assessed using the Comprehensive Feeding Practices Questionnaire (CFPQ) (35). The CFPQ has 49 items on 12 subscales. All items are statements or questions measured on a five-point Likert-type scale ranging from *disagree* to *agree* or from *never* to *often*. The CFPQ has been validated and tested for reliability for parents of children in different age groups (35–38), including in the Norwegian context (39).

Of the 12 subscales, five can be considered healthpromoting feeding practices. We investigated three of these (Cronbach’s *α* values presented are for our sample): *encouraging balance and variety* (e.g. ‘I encourage my child to try new foods’, four items, *α* = 0.47), *shaping a healthy environment* (e.g. ‘Most of the food I keep in the house is healthy’, four items, *α* = 0.68), and *healthy modeling* (e.g. ‘I try to show enthusiasm about eating healthy foods’, four items, *α* = 0.67).

The Cronbach’s *α* values for these subscales were similar to those reported in another study using the same measurements among parents of 1 year olds (36). As Russell et al. did in an Australian study (36), the subscales of two health-promoting feedings practices, *teaching nutrition* and *involvement*, were excluded because of the children’s young age.

### Statistics

In the preliminary analysis, we examined the potential associations between the demographic variables (parental BMI, age, and educational level) and the exposure variables of interest (food choice motives and feeding practices) to assess the need to control for demographic variables in later analyses. No significant associations were found, so no covariates were included.

In the main analysis, we applied the product of coefficients method (40) and tested whether parental food choice motives predicted child’s fruit and vegetable intakes, as well as whether potential associations were mediated by feeding practices. Bootstrapping was performed to estimate the 95% confidence intervals (CI) of the coefficients (*n* = 5,000) (40–42).

**Fig. 1:**
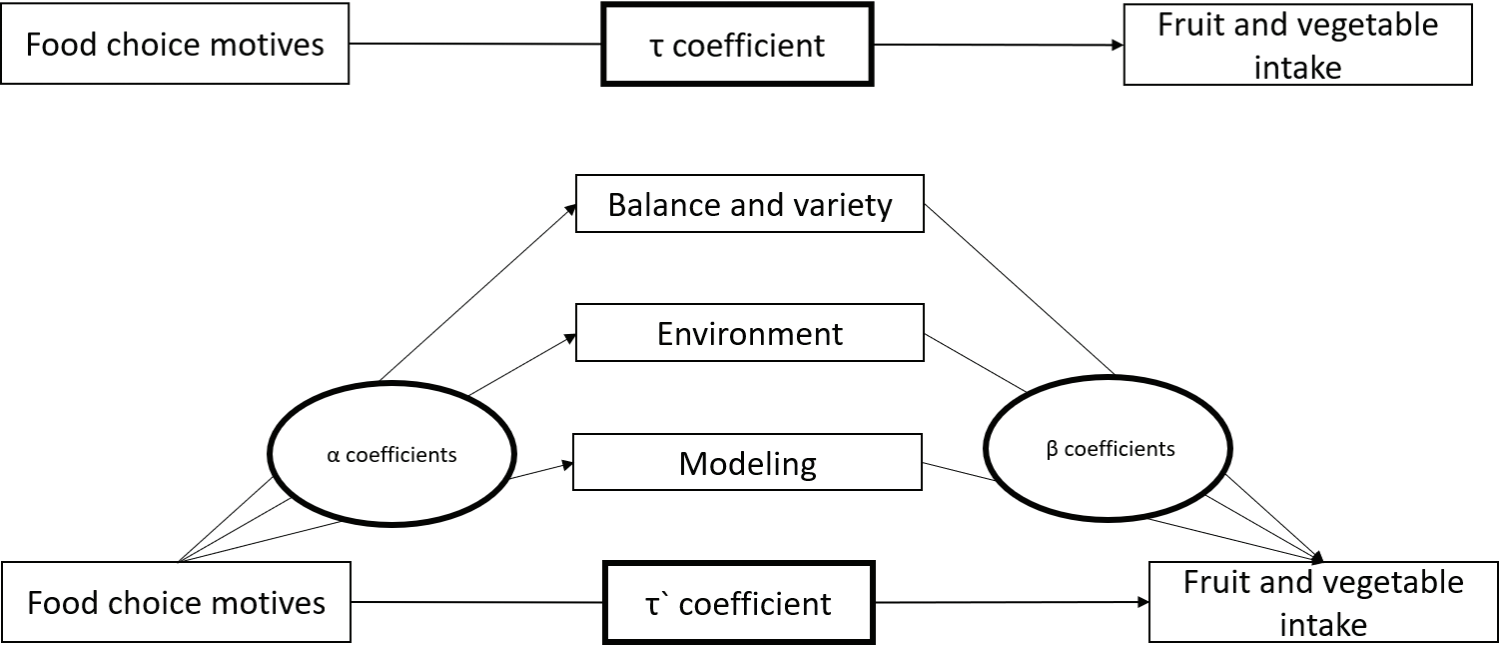
Mediation model of the relations between food choice motives and fruit and vegetable intakes, with three feeding practices as potential mediation factors. Only single mediations were conducted.

Figure 1 shows the investigated associations among food choice motives, infants’ fruit and vegetable intakes, and potential mediation factors.

The overall associations (path *τ*) between food choice motives (predictor variables) and fruit and vegetable intakes (outcome variables) were calculated by regressing the outcome variables on each food choice motive (health, convenience, sensory appeal, price, and familiarity).

The first test in the product of coefficients method is the action theory test, which involves estimating the association between each predictor and the potential mediators (path *α*). Second, the conceptual theory test estimates the association between each potential mediator (health-promoting feeding practices) and the outcome variables, adjusted for the predictor variables (path *β*). The indirect effect was calculated by multiplying the *α*-coefficient by the *β*-coefficient. Bootstrap CIs (*n* = 5,000) were used instead of the SOBEL test (which tests the significance of a mediation effect) to conduct the inferential tests of the indirect effects, as recommended by Hayes (42). To estimate the size of the indirect effect (mediated effect), the ratio of the indirect effect to the total effect was computed by dividing the product of coefficient (*αβ*) by the overall association (*τ*-coefficient) (43). A sample size of 500 is recommended for this estimate, but a smaller sample size is adequate if all estimates are statistically significant (40). In addition, *αβ* and *τ*’ should have the same sign (43). A significant total effect (path *τ*) is not a necessary condition for mediation (42, 44).

All analyses were conducted using IBM SPSS Statistics (Armonk, NY: IBM Corp.), Version 25. Hayes’ Process 3.1 for SPSS (42) was used to perform the single mediation analyses. Data for one person were missing on the FCQ items. The analyses that included the FCQ therefore included data on 297 participants.

## Results

Mean daily servings of fruits and vegetables consumed by the infants were quite high at 2.85 (SD = 1.60) and 3.15 (SD = 1.59), respectively (see Table 2). Food choice motives and feeding practices are also presented in Table 2.

**Table 2:** Mean and standard deviation (SD) for food choice motives (FCQ^a^), health-promoting feeding practices (CFPQ^b^), and infants’ fruit and vegetable intakes

Variables	Mean	SD
FCQ: health	5.15	0.92
FCQ: convenience	5.09	1.08
FCQ: sensory appeal	5.35	0.96
FCQ: price	4.26	1.39
FCQ: familiarity	2.71	1.21
CFPQ: balance and variety	3.57	0.46
CFPQ: environment	3.08	0.78
CFPQ: modeling	3.29	0.69
Fruit intake^c^	2.85	1.60
Vegetable intake^c^	3.15	1.59

a FCQ: Food Choice Questionnaire (measured on a seven-point scale (1–7), ranging from *extremely unimportant* to *extremely important*).

b CFPQ: Comprehensive Feeding Practices Questionnaire (measured on a five-point scale (0–4), ranging from *disagree* to *agree* or from *never* to *often*).

c Daily servings (times/day).

There were high scores on all measured motives except familiarity. In terms of the examined feeding practices, balance and variety (3.57, SD = 0.46) had the highest score.

### Main association between food choice motives and fruit and vegetable intakes (path *τ*)

In our exploration of the relationship between food choice motives and fruit and vegetable intakes, only the motive of health was significantly associated with vegetable intake (*τ* = 0.394, *P* < 0.001) (see Table 3). The effect size was moderate. No other food choice motives were significantly associated with either infants’ fruit or vegetable intakes.

**Table 3:** Associations (*τ* and *τ*′) between food choice motives^a^ (independent variables) and fruit or vegetable intakes (dependent variables) and action (*α*) and conceptual (*β*) theory tests and indirect effects (*αβ*) of health-promoting feeding practices^b^ on the associations between independent and dependent variables

Variables	τ (standard error [SE])	τ*′* (SE)	α (SE)	β (SE)	αβ (SE)	95% confidence interval (CI) for *αβ*^c^	Mediated effect^d^ (%)
**Health^a^**							
*Outcome: fruit intake*	0.073 (0.101)						
Balance and variety		0.027 (0.105)	0.136 (0.028)***	0.334 (0.209)	0.046 (0.028)	−0.007, 0.067	
Environment		0.006 (0.106)	0.268 (0.047)***	0.248 (0.125)*	**0.067 (0.037)**	**0.001, 0.146**	
Modeling		0.072 (0.108)	0.265 (0.041)***	0.004 (0.145)	0.001 (0.037)	−0.073, 0.074	
*Outcome: vegetable intake*	0.394 (0.098)***						
Balance and variety		0.309 (0.100)**	0.136 (0.028)***	0.620 (0.200)**	0.085 (0.031)	0.030, 0.150	**21.4**
Environment		0.289 (0.102)**	0.268 (0.047)***	0.391 (0.120)**	0.105 (0.037)	0.042, 0.186	**26.6**
Modeling		0.329 (0.104)**	0.265 (0.041)***	0.243 (0.140)	0.064 (0.038)	−0.006, 0.143	
**Convenience^a^**							
*Outcome: fruit intake*	−0.110 (0.086)						
Balance and variety		−0.105 (0.086)	−0.013 (0.025)	0.341 (0.201)	−0.005 (0.011)	−0.029, 0.018	
Environment		−0.095 (0.086)	−0.063 (0.042)	0.239 (0.119)*	−0.015 (0.014)	−0.048, 0.007	
Modeling		−0.111 (0.086)	0.030 (0.037)	0.046 (0.135)	0.001 (0.008)	−0.015, 0.018	
*Outcome: vegetable intake*	−0.005 (0.086)						
Balance and variety		0.006 (0.084)	−0.013 (0.025)	0.788 (0.196)***	−0.010 (0.021)	−0.053, 0.031	
Environment		−0.027 (0.084)	−0.063 (0.042)	0.502 (0.116)***	−0.032 (0.023)	−0.082, 0.010	
Modeling		−0.017 (0.085)	0.030 (0.037)	0.400 (0.133)**	0.012 (0.011)	−0.022, 0.049	
**Sensory appeal^a^**							
*Outcome: fruit intake*	0.025 (0.097)						
Balance and variety		0.003 (0.098)	0.063 (0.028)*	0.348 (0.203)	0.022 (0.017)	−0.005, 0.062	
Environment		0.030 (0.097)	−0.017 (0.047)	0.251 (0.119)*	−0.004 (0.013)	−0.035, 0.021	
Modeling		0.021 (0.099)	0.126 (0.041)**	0.032 (0.138)	0.004 (0.020)	−0.035, 0.045	
*Outcome: vegetable intake*	0.121 (0.097)						
Balance and variety		0.072 (0.095)	0.063 (0.028)*	0.768 (0.197)***	**0.047 (0.025)**	**0.005, 0.103**	
Environment		0.129 (0.094)	−0.017 (0.047)	0.502 (0.115)***	−0.009 (0.015)	−0.061, 0.038	
Modeling		0.072 (0.097)	0.126 (0.041)**	0.381 (0.135)**	**0.030 (0.015)**	**0.006, 0.063**	
**Price^a^**							
*Outcome: fruit intake*	−0.088 (0.067)						
Balance and variety		−0.088 (0.067)	0.000 (0.019)	0.349 (0.201)	0.000 (0.008)	−0.017, 0.018	
Environment		−0.082 (0.067)	−0.024 (0.033)	0.244 (0.119)*	−0.006 (0.010)	−0.028, 0.011	
Modeling		−0.089 (0.067)	0.034 (0.029)	0.050 (0.135)	0.002 (0.007)	−0.011, 0.018	
*Outcome: vegetable intake*	0.076 (0.067)						
Balance and variety		0.076 (0.065)	0.000 (0.019)	0.787 (0.195)***	0.000 (0.017)	−0.036, 0.033	
Environment		0.088 (0.065)	−0.024 (0.033)	0.502 (0.115)***	−0.012 (0.017)	−0.047, 0.021	
Modeling		0.062 (0.066)	0.034 (0.029)	0.390 (0.133)**	0.013 (0.013)	−0.009, 0.042	
**Familiarity^a^**							
*Outcome: fruit intake*	0.016 (0.077)						
Balance and variety		0.029 (0.077)	−0.035 (0.022)	0.335 (0.202)	−0.012 (0.012)	−0.040, 0.005	
Environment		0.034 (0.077)	−0.070 (0.038)	0.256 (0.119)*	−0.018 (0.014)	−0.052, 0.003	
Modeling		0.018 (0.078)	−0.035 (0.033)	0.040 (0.136)	−0.001 (0.008)	−0.020, 0.011	
*Outcome: vegetable intake*	0.080 (0.179)						
Balance and variety		0.109 (0.075)	−0.035 (0.022)	0.813 (0.196)***	−0.028 (0.013)	−0.073, 0.007	
Environment		0.116 (0.075)	−0.070 (0.038)	0.518 (0.116)***	−0.036 (0.020)	−0.078, 0.001	
Modeling		0.095 (0.076)	−0.035 (0.033)	0.409 (0.133)**	−0.014 (0.015)	−0.030, 0.011	

* *P* < 0.05

** *P* < 0.01

*** *P* < 0.001.

a Five food choice motive factors (health, convenience, sensory appeal, price, and familiarity) were measured using the Food Choice Questionnaire.

b Three scales of parental practices (encouraging *balance and variety*, shaping a healthy food *environment*, and healthy *modeling*) were measured using the Comprehensive Feeding Practices Questionnaire.

c Bootstrap confidence interval for the indirect effect.

d Estimate of the size of the indirect effect. The ratio of the indirect effect (*αβ*) to the total effect (*τ*): *αβ*/*τ*.

*τ*-Coefficient: estimate of the association between food choice motives and toddler’s fruit or vegetable intakes; *τ′*-coefficient: estimate of the association between food choice motives and toddler’s fruit or vegetable intakes, adjusted for health-promoting feeding practices (mediators); *α*-coefficient: estimate of the association between food choice motives and health-promoting feeding practices (mediators); *β*-coefficient: estimate of the association between health-promoting feeding practices (mediators) and toddlers’ fruit or vegetable intakes, adjusted for food choice motives; *αβ*: product of coefficient estimate, indirect effect.

### Association between food choice motives and potential mediators (path *α*, action theory)

The results from the single mediation analysis are shown in Table 3. The food choice motive of health was significantly associated with all three feeding practices: balance and variety (*α* = 0.136, *P* < 0.001), environment (*α* = 0.268, *P* < 0.001), and modeling (*α* = 0.265, *P* < 0.001). All relationships were in the expected positive direction, with greater values for health food choice motive associated with higher scores on these feeding practices. The food choice motive of sensory appeal was also positively associated with balance and variety (*α* = 0.063, *P* = 0.023) and modeling (*α* = 0.126, *P* = 0.002). The food choice motives of convenience, price, and familiarity were not significantly associated with any of the feeding practices.

### Associations between potential mediators and fruit and vegetable intakes (path *β*, conceptual theory)

As Table 3 reflects, in this single mediation model, path *β* shows the associations between health-promoting feeding practices and fruit and vegetable intakes, adjusted for the food choice motives. When adjusted for the motive of health, the conceptual theory tests revealed that the feeding practice of environment was associated with fruit intake (*β* = 0.248, *P* = 0.001), whereas both environment (*β* = 0.391, *P* = 0.001) and balance and variety (*β* = 0.620, *P* = 0.002) were related to infants’ intake of vegetables.

When adjusted for the other food choice motives (convenience, sensory appeal, price, and familiarity), the tests revealed that environment was associated with fruit intake, whereas all three practices were associated with vegetable intake. All statistically significant relations were positive, such that greater scores on these feeding practices were associated with a higher intake of fruits or vegetables among the infants.

### Mediation effect (path *αβ*)

The feeding practices of balance and variety (*αβ* = 0.085, CI: 0.030–0.150) and environment (*αβ* = 0.105, CI: 0.042–0.186) mediated the relationship between health motive and vegetable intake (Table 3), with a small effect size. This means that the association between health motive and infant’s higher consumption of vegetables was partly explained by the encouragement of balance and variety and by the creation of a healthier food environment. The percentage of the effect mediated was 21.4% for balance and variety and 26.6% for environment.

Despite the lack of a direct significant association between health motive and fruit intake (see Table 3), the feeding practice of environment (*αβ* = 0.067, CI: 0.001–0.146) emerged as a mediator in this relationship. However, the effect size was small.

No significant association was observed between sensory appeal motive and vegetable intake, but balance and variety (*αβ* = 0.047, CI: 0.005–0.103) and modeling (*αβ* = 0.030, CI: 0.006–0.063) mediated this relationship. The effect sizes were again small.

## Discussion

The aim of this study was to examine the associations between parents’ food choice motives and infants’ fruit and vegetable intakes, as well as the mediating effects of parents’ health-promoting feeding practices on these associations. Health was the only motive that was directly associated with a higher infant vegetable intake. No motives were associated with fruit intake. The feeding practice of encouraging balance and variety mediated the association between health motive and vegetable intake and the association between sensory appeal motive and vegetable intake. The associations between health motive and both fruit and vegetable intakes were mediated by the feeding practice of shaping the environment. Modeling was the only mediator of the association between sensory appeal motive and vegetable intake.

### Food choice motives and fruit and vegetable intakes

The importance of health as a food choice motive for older children has been assessed in other studies, for example, two studies of preteens in Nordic countries (45, 46), which have shown a pattern similar to that found in the present study.

Studies conducted in the United States have reported an association between the motive of health and fruit and vegetable intakes among preschoolers (21) and among 7- to 11-year-old children (24). In the US context, an association between the motive of natural content and fruit and vegetable intakes has also been reported among preschoolers (21). One study also assessed convenience as a motive, finding a negative association of this motive with both fruit and vegetable intakes (24). Roos et al. (46) assessed whether food choice motives predicted a higher intake of ‘nutrient-dense food’ (fruits, vegetables, berries, and rye bread) among 10- to 12-year-old Finnish children. They reported that parental motives of health and nutrient content and sensory appeal were positively associated with healthy food intake, and that the motive of convenience was negatively associated with nutrient-dense food intake.

An Australian study targeting parents of 2- to 5-year-old children reported a tendency for the motive of health and nutrition to be associated with children’s fruit and vegetable liking (22). The parents in the same study rated health and nutrition factors as the most important motive when choosing food for their children. Nevertheless, the children’s own food preferences and requests influenced the children’s food decisions to a larger degree than did the parents’ health motive. The present research was the first study to assess these relationships in infants, and our results are in line with the existing literature on the importance of the health motive.

### Food choice motives and parental feeding practices

In terms of the direct association between the parental food choice motives and feeding practices, parents with higher scores on the motive of health in the present study also had higher levels of all assessed health-promoting feeding practices, indicating the importance of this motive. It is not surprising that parents with an interest in health would use health-promoting feeding practices, but previous work has shown that healthy motives do not always translate into beneficial actions such as shaping a healthy food and eating environment for children (23). It has been recommended that parents serve as positive role models by creating a supportive home environment through increasing their encouragement of healthy eating, making fruits and vegetables more available, and incorporating rules to govern eating behavior (47, 48). According to Pollard et al. (30), sensory appeal (e.g. taste, texture, smell, and appearance) can influence which foods a person chooses to buy and consume. In the Food4toddlers study, a high parental score on sensory appeal motive was not associated with a higher fruit or vegetable intake, but it was associated with two of the three health-promoting feeding practices: healthy modeling and encouraging balance and variety.

### The mediating effect of health-promoting feeding practices

Regarding the mediation effects, balance and variety and environment were stronger mediators, compared with modeling. The effect sizes were small, but, for both environment and balance and variety, the mediation effects explained more than 20% of the effect of health motive on vegetable intake, meaning that these practices partly explained the association.

The feeding practice of shaping a healthy environment seemed to be an important mechanism between health motive and the quantity of fruits and vegetables children ate (49, 50). Corsini et al. (51) recommended focusing on shaping a healthy environment instead of on restrictive practices (i.e. coercive control practices). A recent review on how to reduce parents’ provision of unhealthy foods to 3- to 8-year-old children recommended more research on the effects of persuasion, modeling, and environmental restructuring (52).

The other mediator shown to be important in the present study was encouraging balance and variety, meaning that the parent encourages the child to eat new and varied foods and talks positively about healthy foods. A study of 3- to 5-year-old children (53) and another study of 6- to 18-year-olds (48) showed that this type of parental encouragement positively influenced both fruit and vegetable consumption, in contrast to a Norwegian study of preteens (50) that did not find balance and variety to be associated with either fruit or vegetable consumption.

Healthy modeling may contribute to higher fruit and vegetable intakes (54). However, in the present study, healthy modeling did not mediate the associations between food choice motives and fruit and vegetable intakes to the same degree as the other examined feeding practices. The young age of the children in our study may explain the lack of mediating effects for this practice because very young children may not recognize what their parents eat or take notice of the link between their parents’ engagement with healthy foods and the food offered to the children.

The assessed feeding practices mediated the associations between food choice motives and vegetable intake to a larger extent than they did the associations with fruit intake. This supports the notion that fruits and vegetables should be treated as separate entities in new interventions, as recommended by Glasson et al. (55) and Appleton et al. (56).

To our knowledge, the present study was the first to explore the associations between food choice motives, health-promoting feeding practices, and fruit and vegetable intakes in this age group. Two studies conducted in the United States (21, 24) explored the same overall constructs (feeding motives, child’s diet, and parental feeding practices), but these studies examined negative feeding practices, other diet outcomes, and only two food choice motives in each article (compared with the five treated in the present study). The results of these studies were not consistent. Their first study showed that the children (aged 3–6 years) of parents who used negative feeding practices were often more likely to eat unhealthy foods, despite their parents’ healthy feeding motives (21). The second study found that children (aged 7–11 years) whose mothers emphasized health motives consumed more healthy food and less unhealthy food; however, in contrast to the results of the first study, negative feeding practices did not mediate the associations in this second study (24). The children’s age difference between the two studies may explain the different results. The children in both studies were older than those in our study. Because dietary habits are established early and track into adolescence, focusing on the youngest age groups is important from a public health perspective.

### Strengths and limitations

A potential strength of this study is that, by using social media (Facebook) as a recruitment channel, participants from the entire country (57) could be included, and we were able to reach a relatively large sample of children born in a restricted time frame. Additionally, the questions used in the study were validated and reliability tested and have been widely used in other studies. Finally, the study is particularly important because there is a lack of studies on this young age group (28).

An important limitation of this study involves its cross-sectional design, which hindered causal interpretation of the findings (58). Some questionnaires were, unfortunately, not fully answered, probably because of the length, and we included those who had answered more than half of the questionnaire. We wanted to recruit a broad spectrum of parents using Facebook, which is known to be an effective recruitment arena (59). However, not reaching non-users of Facebook is a limitation in our study. The aim was to reach more fathers and parents with low Socioeconomic status (SES) than would otherwise be possible; however, the majority of people recruited were mothers (98.0%) with high SES. It is not known whether the findings would have been different if more fathers had participated. It is possible that using video services (such as YouTube), as recommended in a recently published Norwegian study (60), would have been a better approach. Another potential limitation is that parents may have reported a healthier lifestyle than they actually followed because they may have been ashamed of some of their choices, as has been seen in comparable studies (61).

Regarding the generalizability of our findings, participants were more highly educated, compared with national figures (62). In addition, the included parents were more likely to be especially interested in health and nutrition issues because they initially responded to the advertisement on Facebook. A more representative study sample might have given different results according to infant diet, which studies in Europe (63) and Australia (64) have indicated. Finally, our findings contribute to the knowledge of parental determinants (or predictors) of fruit and vegetable consumption among infants, but they should not be generalized to other age groups.

## Conclusion

Our results confirm previous findings on the importance of health motives for infant and children’s vegetable consumption. We also see that the health-promoting feeding practices assessed mediate associations between some food choice motives (health and sensory appeal) and fruit and vegetable consumption, but not to a large degree.

Health-promoting feeding practices may mediate associations between parental characteristics other than food choice motives, such as knowledge, attitudes, and general parenting style, and infants’ fruit and vegetable intakes. Such associations should be examined in further studies to identify which feeding practices and potential predictors of these feeding practices should be targeted in interventions to enhance the intake of fruits and vegetables among infants and toddlers.

Our results contribute to understand the underlying motives of parental feeding behaviors in this age group. The results may be different investigating older children because, for example, the family interaction and tastes changes by age. Continuing to study the interplay between infant’s food intake and parents’ motives and practices about healthy and unhealthy eating behaviors is an important endeavor.

## Authors’ contributions

NCØ, ERH, and FNV conceived the study. MR, ERH, FNV, and NCØ initiated and designed the study and developed the intervention. MR performed the data collection, supervised by ERH, FNV, and NCØ. MR performed the analysis, supervised by WVL and NCØ. MR drafted the manuscript with substantial input from ERH, FNV, WVL, and NCØ. All authors contributed to read and approved the final version of this manuscript.

## Conflict of interest and funding

The authors declare that they have no competing interests. This study is funded by the University of Agder. The financial contributor was not involved in designing the study, collection, analyses, and interpretation of data or in writing the manuscript.
